# 2783. Evaluation of Appropriate Antibiotic Prescribing Following Modification of Culture & Susceptibility Cascade for High Risk AmpC-Producing Gram-Negative Bacteria

**DOI:** 10.1093/ofid/ofad500.2394

**Published:** 2023-11-27

**Authors:** Andy Lim, Terrence D McSweeney, Phyu Thwe, Mei Chang, Hongkai Bao, Philip J Lee, Yi Guo

**Affiliations:** Montefiore Medical Center, Flushing, New York; Montefiore Medical Center, Flushing, New York; Montefiore Medical Center, Flushing, New York; Montefiore Medical Center, Flushing, New York; Montefiore Medical Center, Flushing, New York; Children's Hospital at Montefiore, Bronx, New York; Montefiore Medical Center, Flushing, New York

## Abstract

**Background:**

Updated cascade reporting of antimicrobial susceptibility (CRAS) for high-risk AmpC-producing Gram-negative organisms was implemented by the antimicrobial stewardship (ASP) and microbiology teams in June 2022 based on recent IDSA guidance. The CRAS focuses on highlighting weak inducers and poor substrate antibiotics such as cefepime and meropenem for AmpC-producing organisms: *H. alvei, E. cloacae, C. freundii, K. aerogenes, and Y. enterocolitica* (HECK-Yes).

**Methods:**

A retrospective, observational, quasi-experimental study was conducted comparing the appropriateness of antimicrobials prescribed for infections with HECK-Yes organisms between pre-implementation period (July 2021-April 2022) and post-implementation period (July 2022-April 2023). Patients >18 years old with a positive blood or respiratory culture with any HECK-Yes organisms were included. The primary outcome of the study was the frequency of patients on appropriate antibiotics within 24 hours of final susceptibility report. Secondary outcomes included time to appropriate antibiotic order and administration, clinical success, microbiological failure, mortality, and reinfection/readmission within 30 days.

**Results:**

Fifty patients were included in the pre- and post-implementation groups. Baseline characteristics were similar between the two groups (Table 1). A relative increase of 38.7% was observed in the appropriateness of antibiotics within 24 hours of final culture & susceptibility report (pre: 62% vs. post: 86%, p = 0.01). No significant difference was observed between the two groups in time to appropriate antibiotic order (pre: 4.8 hours vs. post: 3.3 hours, p = 0.37) or administration (pre: 5.9 hours vs. post: 8.0 hours, p = 0.53). There was a trend towards a lower 30-day mortality rate in the post-implementation group (pre: 24% vs. post: 14%, p = 0.10). No significant difference was observed for clinical success, microbiological failure, and reinfection/readmission within 30 days (Table 2).

**Figure:**
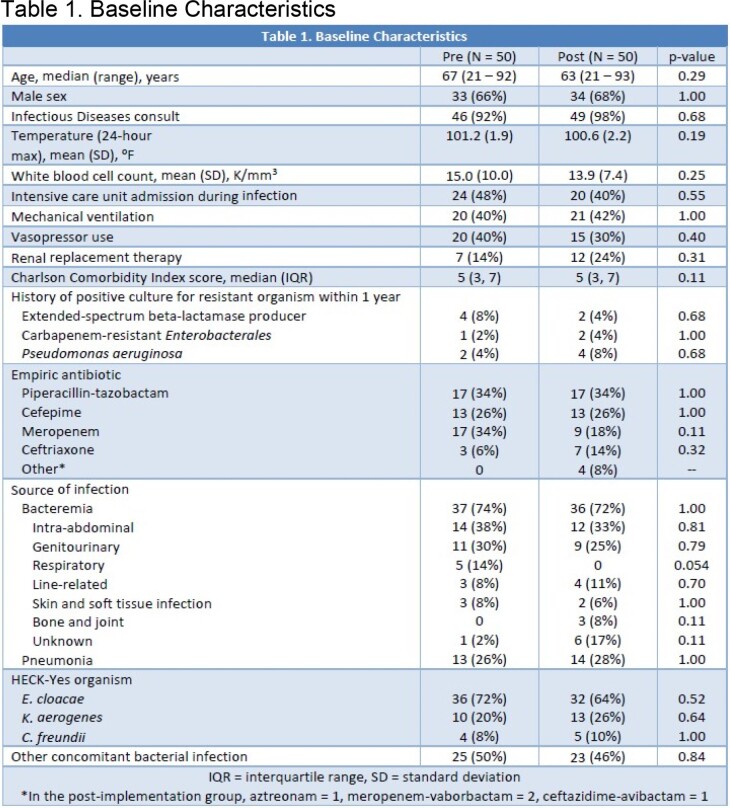

**Figure:**
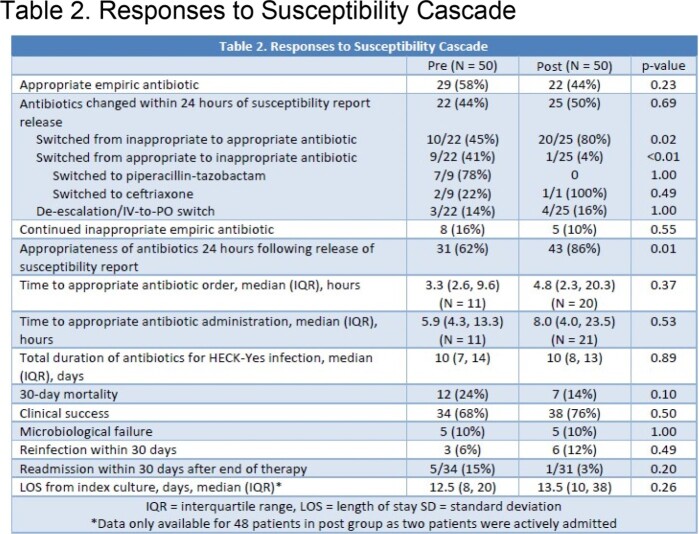

**Conclusion:**

Updating CRAS effectively increased the frequency of appropriate antibiotic prescribing for the treatment of HECK-Yes infections. The collaborative efforts of ASP and microbiology teams optimized antimicrobial prescribing for infections with HECK-Yes organisms at our institution.

**Disclosures:**

**All Authors**: No reported disclosures

